# Esophageal squamous cell carcinoma with colonic and rectal metastases: a rare case report

**DOI:** 10.3389/fonc.2025.1519922

**Published:** 2025-04-08

**Authors:** Jianquan Yang, Wen Guo, Xuezhou Pang, Yuerong Tang, Yakun Zhang, Beilei Zeng, Yan Gui, Daiyuan Ma

**Affiliations:** ^1^ Department of Oncology, Affiliated Hospital of North Sichuan Medical College, Nanchong, Sichuan, China; ^2^ Pharmacy Department, Affiliated Hospital of North Sichuan Medical College, Nanchong, Sichuan, China; ^3^ Department of Nuclear Medicine, Affiliated Hospital of North Sichuan Medical College, Nanchong, Sichuan, China

**Keywords:** esophageal squamous cell carcinoma, rectal metastasis, colon metastasis, treatment, case report

## Abstract

As a common malignant tumor, esophageal cancer is easy to relapse and distant metastasis, and the prognosis is very poor. Colon and rectal metastasis of esophageal cancer is extremely rare. This study reports a case of colorectal and rectal metastasis in an esophageal squamous cell carcinoma patient. The patient was a 65-year-old man who presented with progressive swallowing obstruction. Gastroscopy and pathological biopsy revealed low-differentiated squamous cell carcinoma in the lower esophagus (32cm from the incisor). After completing the relevant examination, the patient was evaluated by the thoracic surgeon and showed no indication of surgery. Then the patient was received 2 cycles of Abraxane plus cisplatin with Sintilimab. After the treatment, the esophageal lesion was examined by Chest CT, and assesses again by the surgeon again and radical radiotherapy was recommended without indication of surgery. After radiotherapy, the patient underwent comprehensive imaging examination. Abdominal CT showed mass in the lower abdomen. Colonoscopy and pathological biopsy showed squamous cell carcinoma of colon and rectum. According to the pathological type and tumor monism, and communication with the pathologist, the patient was diagnosed to be esophageal cancer with rectal and colon metastasis. Through this case report, we hope to deepen the understanding of rare esophageal squamous cell metastasis, and comprehensive examination should be conducted before initial treatment to evaluate the tumor status.

## Introduction

Esophagus cancer is a common malignant tumor of digestive system. According to CLOBOCAN 2024, there are about 600,000 new cases of esophageal cancer in the world, and about 540,000 deaths, while nearly half of the global incidence and death cases of esophageal cancer occur in China (1). At the same time, the incidence and mortality of esophageal cancer in China are ranked sixth and fourth respectively in the incidence and death of malignant tumors in China, posing a serious threat to human health (2). The epidemiology of esophageal cancer shows that the distribution of esophageal cancer patients in China is geographically distributed, which may be related to the living habits of patients, mainly including hot food and pickled food (3, 4). Esophageal cancer patients have no obvious symptoms in the early stage of the disease. When esophageal fistula occurs, patients may have fever, cough, sputum and other symptoms (5, 6). The common distant metastasis of esophageal cancer includes supraclavicular lymph node metastasis and metastasis of liver, lung, brain, bone, but the metastasis of rectum, colon is very rare. We report a rare case of rectal and colonic metastasis of lower esophageal squamous cell carcinoma.

## Case presentation

All procedures performed in human participants met the ethical standards of the institutional and/or national research committee(s) and with the Helsinki Declaration (as revised in 2013). Written informed consent was obtained from the patient.

**Figure 1:**
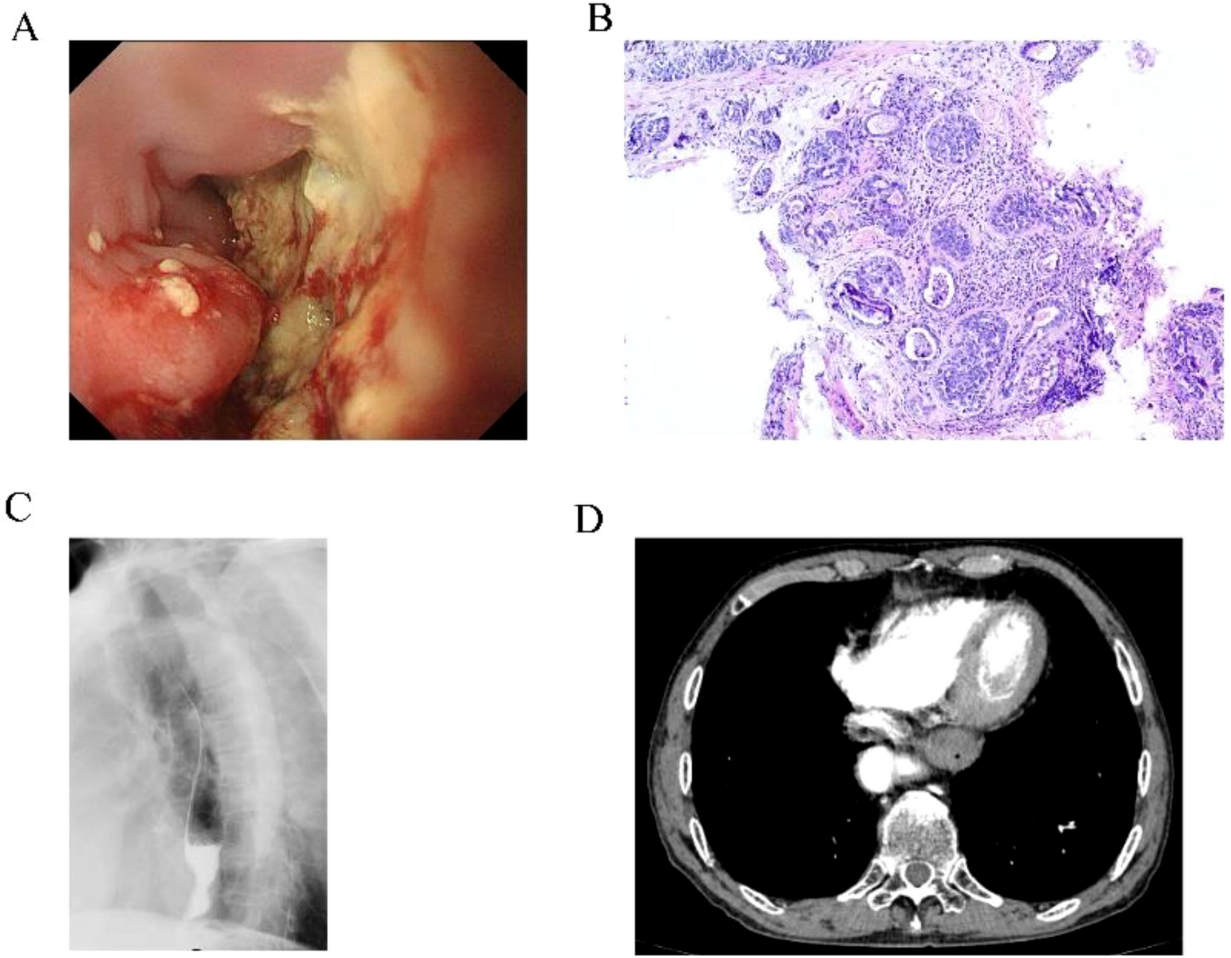
Pre-treatment examination results of the patients. **(A)** Gastroscopy results showed neoplasm in the lower esophagus. **(B)** The lesion site of the esophagus showed squamous cell carcinoma. **(C)** Gastrointestinal angiography showed stenosis of the lower esophagus. **(D)** Chest CT showed thickening of the lower wall of the esophageal tube with stenosis.

**Figure 2:**
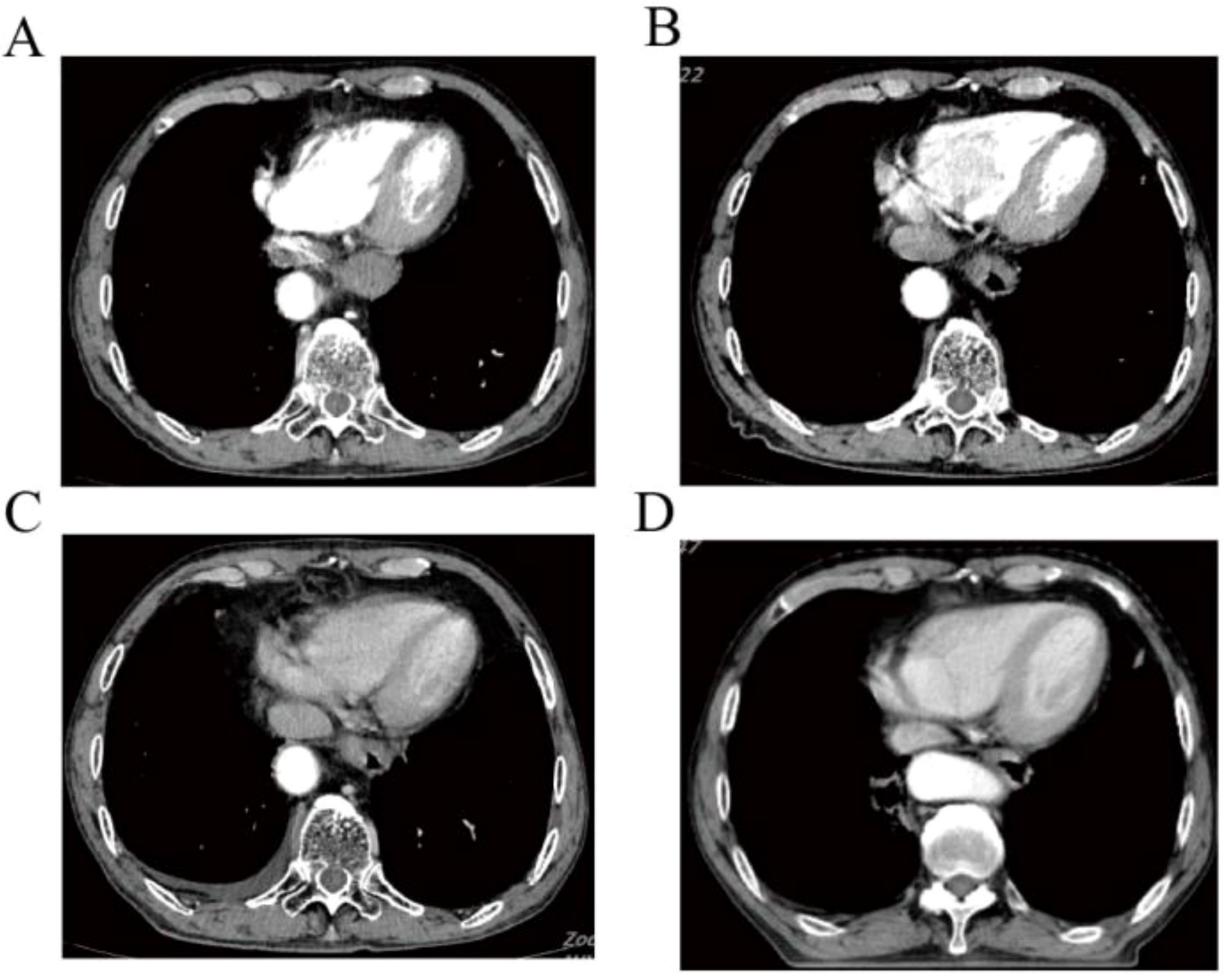
Chest CT of shows the changes of esophageal lesions of anti-tumor therapy. **(A)** Esophageal lesions before treatment. **(B)** Esophageal lesions after 2 cycles of chemotherapy combined with immunotherapy. **(C)** Esophageal lesions before radiotherapy. **(D)** Esophageal lesions on chest CT were reexamined after radiotherapy.

**Figure 3:**
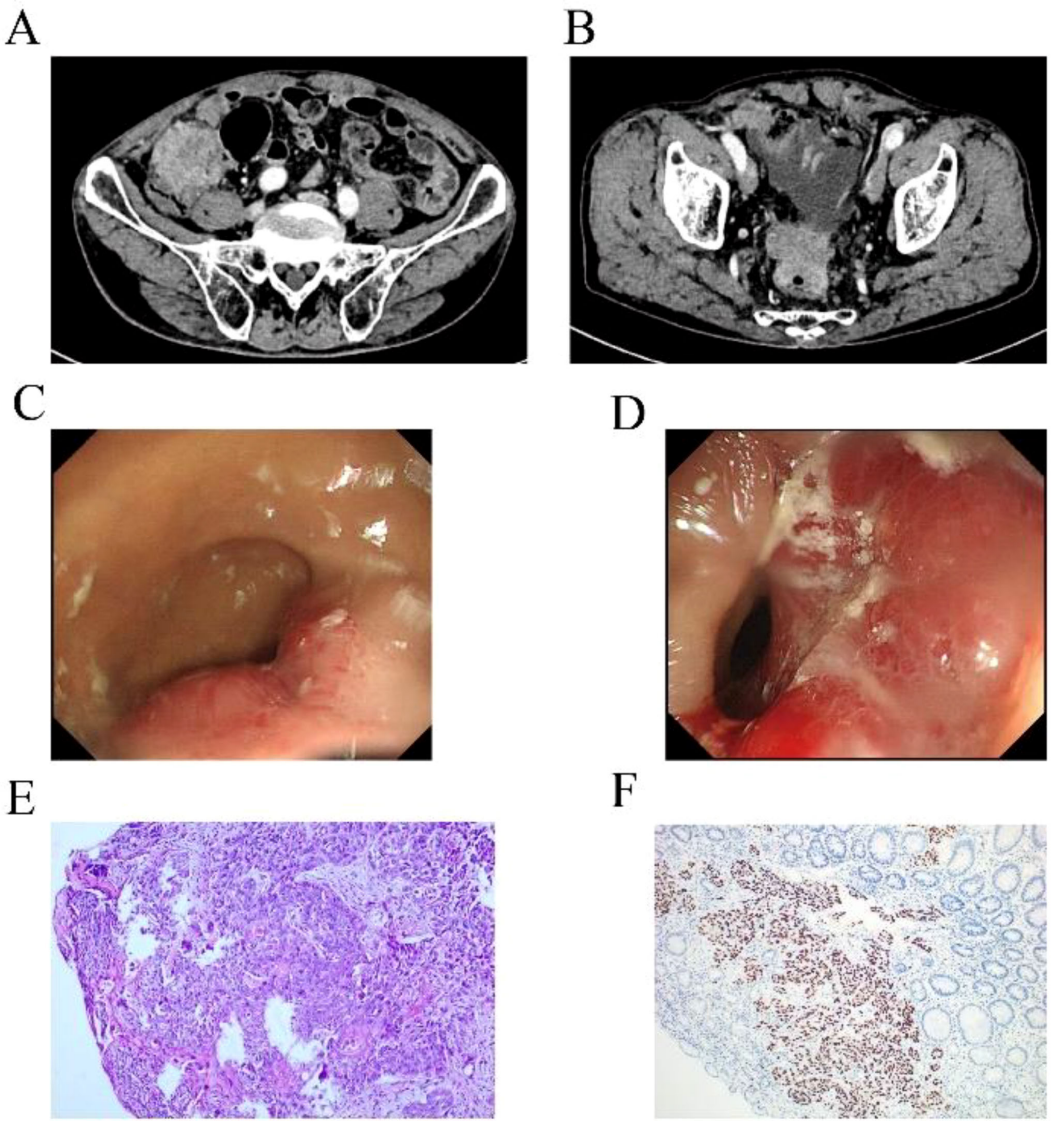
Examination results of rectal and colon metastases. **(A, B)** Abdominal CT showed space-occupying lesions of colon and rectum. **(C, D)** Colonoscopy of colon and rectum found new organisms. **(E, F)** HE staining and immunohistochemical results supported squamous cell carcinoma.

**Figure 4:**
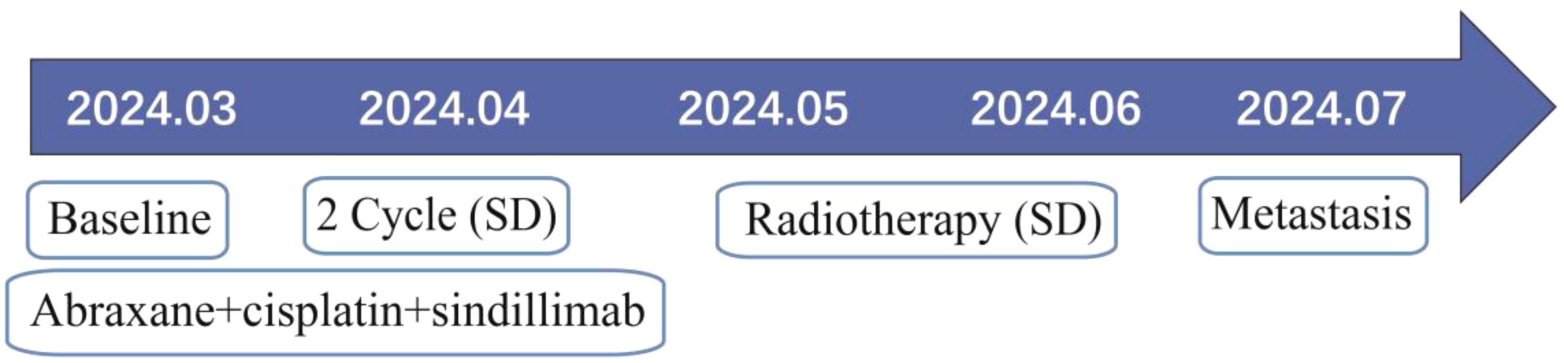
The antitumor treatment timeline.

The patient was a 67-year-old man who presented to the hospital in March 2024 with progressive swallowing obstruction for more than 1 month. Gastroscopy was performed on the patient, and new organisms were found in the esophagus 33-37 cm away from the incisor. The lesions occupied more than 2/3 of the lumen, resulting in stenosis of the lumen (**Figure 1A**). Esophageal pathological examination of the patient revealed esophageal squamous cell carcinoma (**Figure 1B**). Further, the patient underwent upper gastrointestinal angiography, which showed that irregular filling defects were observed in the lower esophagus (**Figure 1C**). The results of chest CT showed that the wall of the lower esophagus was significantly thickened, about 1.8 cm at the thicker part, and the surrounding fat space was blurred. A mass soft tissue density shadow was observed in the hepatogastric space, which was indistinctly separated from the lower esophagus, with a size of 4.9×2.3 cm (**Figure 1D**). After assessed by the thoracic surgeon, the patient had no indications for surgery and received neoadjuvant chemotherapy and immunotherapy. The patients were treated with 2-cycle TP (Abraxane 400 mg, d1, cisplatin 120 mg, d1) combined with sindillimab (200mg, d1) on 2024-03-28 and 2024-4-18 respectively. Chest CT reexamination showed that the lower esophageal wall was thickened, with the thicker part about 1.2 cm, and the surrounding fat space was blurred. In the hepatogastric space, there was a dense mass of soft tissue with an unclear boundary between the lower end of the esophagus and the adjacent gastric wall, with a size of 3.7×1.9 cm (**Figures 2A, B**). After 2 cycles of chemotherapy combined with immunotherapy, the esophageal lesions and lymph nodes in the hepatogastric space were all reduced, but the fatty space around the esophagus was still fuzzy. According to the evaluation of the surgeon, the patient had no operation opportunity. 2024-04-29, the patient received radiotherapy with a dose of P-GTV 50 Gy/25 Fx for esophageal tumor lesions and hepatic and gastric space lymph node lesions(**Figure 2C**). The completion time of radiotherapy was 2024-06-09. 2024-07-07, the patient returned to the hospital and underwent upper gastrointestinal angiography, and the results showed that irregular and mild stenosis about 1.1cm in length was observed in the lower thoracic section of the esophagus, and the expansion was slightly limited, and the passage of contrast agent was not significantly hindered. Chest CT showed that the wall of the lower esophagus was significantly thickened, about 1.3cm at the thicker part, and the surrounding fat space was blurred. Multiple nodules were found in the hepatogastric space, which was indistinct from the lower esophagus (**Figure 2D**). After admission, the patient underwent abdominal CT scan due to the symptoms of blood in stool for nearly 1 month. Abdominal CT results showed a 4.9 x 4.4 cm mass in the right lower abdomen, which was closely related to the ascending colon, and the rectovesical space lesions were closely related to the adjacent anterior rectal wall (**Figures 3A, B**). Further, the colonoscopy results showed that the neoplasm was found at 10 cm and the lumen was narrowed by the tumor. The neoplasm was found at 70 cm (ascending colon) and the lumen was narrowed significantly (**Figures 3C, D**). Pathological results showed that: “Ascending colon 70 cm” and “rectum” check see carcinoma, immunohistochemistry, CK5/6 (+), about (+), P40 (+), Ki67 (about 40%) +, CK7 (-), CK20 (-), CDX - 2 (-), SATB2 (-), ascending colon and rectum saw squamous cell carcinoma (**Figures 3E, F**). Based on the antitumor treatment timeline (**Figure 4**) and the examination results of the patient, the diagnosis of lower esophageal squamous cell carcinoma with distant metastasis was clear. Since the patient had incomplete intestinal obstruction accompanied by hematochezia, it was recommended to perform tumor reduction for the lesions of rectum and colon after the evaluation of the surgeon. Unfortunately, after communicating with the patient’s family, the patient and the patient’s family finally chose not to undergo surgery and chemotherapy after considering the patient’s condition and economic situation, and returned to the local hospital for palliative care.

## Discussion

Due to the deep anatomical structure and hidden onset, most patients with esophageal cancer have been locally advanced or advanced at the time of clinical treatment. The metastasis of esophageal cancer can be divided into lymphatic metastasis, blood metastasis and direct spread metastasis (7). Studies have shown that about 20 percent of patients develop distant metastases at first visit (8). When patients develop distant metastases, the disease progresses rapidly, the treatment is poor, the prognosis is poor, and the median time to formation is only 3-5 months (9). The common distant metastasis organs of esophageal cancer are mainly lung, liver and bone (6). Metastasis of esophageal cancer to the rectum and colon is rare (10, 11), malignant bowel obstruction (MBO) is even rarer. MBO is a common end-stage event in patients with advanced tumors. Studies have shown that the incidence of MBO in advanced tumors is 5% to 43%, with the highest risk of ovarian cancer (5% to 51%) and gastrointestinal tumors (10% to 28%) (12, 13). The average time from cancer diagnosis to MBO was 14 months (14). For patients with MBO, the goal of treatment is to improve the quality of life and prolong survival. In this case, MBO was diagnosed by abdominal CT and colonoscopy after the appearance of blood in stool. After general surgical consultation, palliative tumor reduction is recommended. For MBO patients, whether to perform surgery is still controversial. Although studies have shown that MBO patients receiving surgery compared to conservative treatment, patients do not benefit significantly (15). However, the study of Reed et al. (16) showed that compared with conservative treatment, surgical treatment can prolong the survival time of patients (15 months VS 3 months). The National Comprehensive Caner Network (NCCN) recommends systemic palliative chemotherapy plus immunotherapy for patients with stage IV esophageal squamous cell carcinoma, combined with local therapy if necessary (17). After standard first-line chemotherapy combined with immunotherapy, the esophageal primary lesion was controlled, but the colorectal metastasis site advanced, and tumor reduction surgery was recommended. Unfortunately, the patient refused to undergo tumor reduction surgery and was discharged automatically.

The mechanism of rectal metastasis of esophageal cancer remains unclear. It has been suggested that lymphatic metastasis is one of the possible causes, because of the abundance of lymph in the esophagus, tumor cells may retrograde through the lymphatic system to the colorectal, resulting in colorectal metastasis (11, 18).

Traditional imaging methods for clinical evaluation of esophageal cancer patients with distant metastasis mainly include ultrasound, CT and MRI (19, 20). It has been reported that the sensitivity of CT in the diagnosis of distant metastasis of esophageal cancer is only 37% ~ 66%, which is largely dependent on pathological diagnosis. Although MRI is more sensitive than CT in the external invasion of esophageal cancer, it is not better than CT in the preoperative staging of esophageal cancer. Conventional imaging is based on histomorphological changes and is a local examination. positron emission computed tomography/computed tomography (PET/CT), PET/CT integrates molecular imaging function and anatomical structure imaging, which can reflect the morphological characteristics and metabolic status of the lesion, and show the heterogeneity and treatment-induced changes within the tumor in a non-invasive manner. PET/CT examination makes up for the above shortcomings, it can detect functional metabolic abnormalities earlier before the lesion morphological changes, and has its absolute advantages in the diagnosis of distant metastasis of esophageal cancer (20). Since pelvic imaging examination is not a routine examination item for esophageal cancer, pelvic imaging examination was lacking when the patient was first diagnosed. After the occurrence of hematochezia in patients, relevant examinations were improved to finally determine the colon and rectal metastasis of esophageal cancer. From the perspective of the diagnosis and treatment process of this patient, PET/CT examination of tumor patients at the first diagnosis can find uncommon tumor metastasis sites, and provide more information for tumor staging and treatment plan.

## Conclusions

To our knowledge, this is only the first reported case of esophageal squamous cell carcinoma with colonic and rectal metastasis. From the diagnosis and treatment of this patient, we need to learn the following lessons: 1. For newly diagnosed patients with esophageal cancer, complete imaging examination, especially PET/CT, is of great significance to find rare tumor metastasis and accurate tumor staging. 2. Multi-disciplinary treatment (MDT) is very necessary, and the implementation of MDT can enable patients to get timely, standardized and effective treatment. 3. Strengthen doctor-patient communication and establish patients’ confidence in treatment.

## Data Availability

The original contributions presented in the study are included in the article/supplementary material. Further inquiries can be directed to the corresponding authors.
